# Polymeric Nanoparticle-Mediated Gene Delivery for Lung Cancer Treatment

**DOI:** 10.1007/s41061-017-0128-5

**Published:** 2017-03-13

**Authors:** Narsireddy Amreddy, Anish Babu, Ranganayaki Muralidharan, Anupama Munshi, Rajagopal Ramesh

**Affiliations:** 1 Department of Pathology, Stanton L. Young Biomedical Research Center, University of Oklahoma Health Sciences Center, Suite 1403, 975 N.E., 10th Street, Oklahoma City, OK 73104 USA; 2 Department of Radiation Oncology, University of Oklahoma Health Sciences Center, Oklahoma City, OK USA; 3 Stephenson Cancer Center, University of Oklahoma Health Sciences Center, Oklahoma City, OK USA; 4 Graduate Program in Biomedical Sciences, University of Oklahoma Health Sciences Center, Oklahoma City, OK USA

**Keywords:** Lung cancer, Gene therapy, Polymer nanoparticles, Receptors, Targeted delivery

## Abstract

In recent years, researchers have focused on targeted gene therapy for lung cancer, using nanoparticle carriers to overcome the limitations of conventional treatment methods. The main goal of targeted gene therapy is to develop more efficient therapeutic strategies by improving the bioavailability, stability, and target specificity of gene therapeutics and to reduce off-target effects. Polymer-based nanoparticles, an alternative to lipid and inorganic nanoparticles, efficiently carry nucleic acid therapeutics and are stable in vivo. Receptor-targeted delivery is a promising approach that can limit non-specific gene delivery and can be achieved by modifying the polymer nanoparticle surface with specific receptor ligands or antibodies. This review highlights the recent developments in gene delivery using synthetic and natural polymer-based nucleic acid carriers for lung cancer treatment. Various nanoparticle systems based on polymers and polymer combinations are discussed. Further, examples of targeting ligands or moieties used in targeted, polymer-based gene delivery to lung cancer are reviewed.

## Introduction

Lung cancer is the leading cause of cancer-related mortality in both men and women [1]. Two main subtypes of lung cancer exist: (1) non-small cell lung cancer (NSCLC) and (2) small cell lung cancer (SCLC). NSCLC accounts for about 85% of lung cancers, with the remaining 15% characterized as SCLC. NSCLC has three subtypes: (1) adenocarcinoma, (2) squamous cell carcinoma, and (3) large-cell carcinoma. Approximately 40% of lung cancers are adenocarcinomas, which originate in the peripheral lung tissue. Twenty-five percent of lung cancers are squamous cell carcinomas, which originate from proximal airway epithelial cells; large cell carcinoma originating from epithelial cells accounts for 15% of lung cancer cases [2].

The conventional treatment methods for lung cancer are surgery, radiotherapy, and chemotherapeutics [3, 4]. These treatment methods have some limitations because of their poor therapeutic efficiency, non-specific interactions, and toxicity to normal tissues [5]. Gene therapy is an alternative approach that can improve therapeutic efficiency and reduce toxicity to normal tissues [6, 7].

In cancer therapy, RNAi has been recognized as an efficient method of targeted therapy that is facilitated through target-specific oligonucleotides that knock down expression of the genes [8]. RNAi can be achieved using small interfering RNA (siRNA), short hairpin RNA (shRNA), and micro RNA (miRNA), which has an average of 21–23 base pair oligonucleotides [9]. The targeted delivery of genes into cancer cells results in the specific silencing of genes that are actively involved in tumor growth, angiogenesis, and metastasis [10, 11]. For successful and efficient gene delivery, oligonucleotides need carrier support to transfect into cells because of the possibility of degradation by nucleases while circulating and in the harsh conditions of cellular endo-lysosomes [12]. Nanoparticles are promising carriers for transfecting genes into cancer cells. Among the different types of nanoparticles, polymer-based nanoparticles are widely used for gene delivery [13].

Most polymer-based nanoparticles exhibit a positive surface charge on the periphery, which is utilized for electrostatic adsorption and condensation of nucleic acids [14, 15]. Synthetic and natural polymers of different architecture can form nano- or micro-sized particles, depending on the chemical methods used for synthesis [16]. Biocompatibility and biodegradability are important parameters that must be considered when polymers are chosen for gene delivery vehicle fabrication. Moreover, many polymers used in gene delivery systems actively exhibit a “proton sponge effect” that initiates the endo-lysosomal escape of therapeutic gene molecules into the cytoplasm [17]. Further, the polymer’s surface functionality also plays an important role in conjugating biomolecules for therapeutic targeting into cancer cells [18]. The specific delivery into cancer cells is an important strategy to improve therapeutic efficacy and reduce toxicity in normal tissues.

Ligand-based targeting is more promising than other passive and physical targeting methods for gene delivery. Ligands attached to the surface of nanoparticles specifically interact with overexpressed cell surface receptors [19, 20]. Various receptors, including folate receptor alpha (FRA) [21], epidermal growth factor receptor (EGFR) [22], integrins [23], CD44 [24], and transferrin [25], are known to be overexpressed in lung cancer cells. These receptors can be targeted by conjugating folic acid, EGFR antibody or antibody fragments, RGD peptide, hyaluronic acid (HA), and transferrin protein or antibody.

This review discusses different kinds of nanoparticles that are fabricated with artificial or natural polymers for gene delivery in cancer therapy. In addition, we highlight various receptor-targeting strategies that use polymer nanoparticles modified with ligands or moieties for the specific delivery of gene therapeutics.

## Types of Polymeric Nanoparticles Used for RNAi in Lung Cancer

**Fig. 1:**
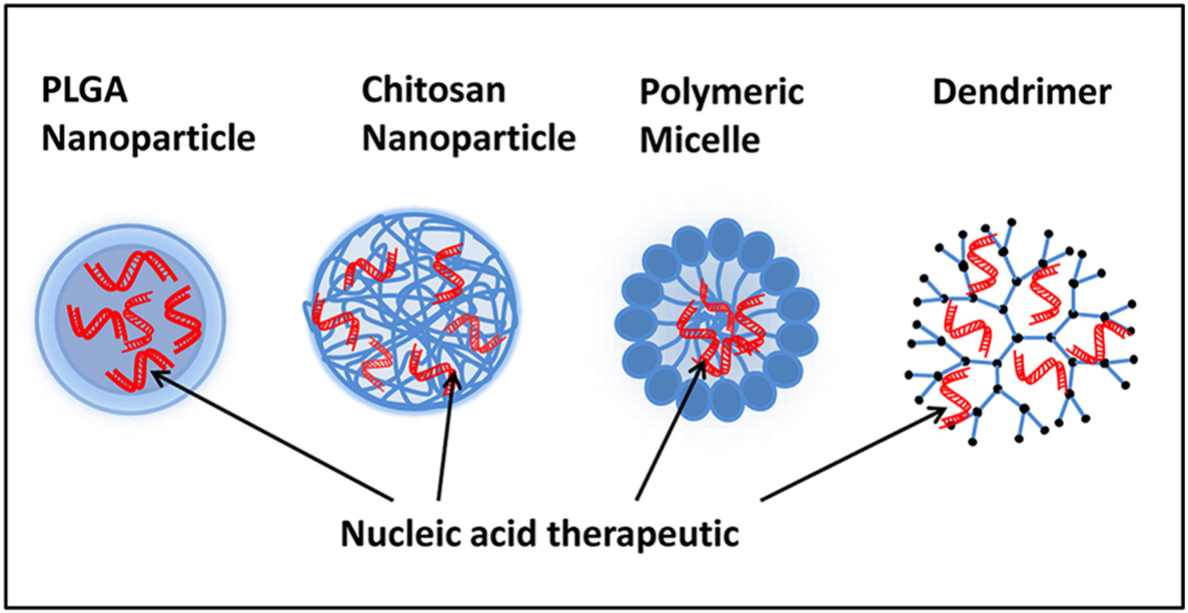
Schematic representation of commonly used polymeric nanoplatforms for gene delivery such as PLGA and chitosan nanoparticles, polymeric micelles, and dendrimers encapsulated with nucleic acid therapeutics for lung cancer treatment

Polymeric nanoparticles are more widely used in gene delivery systems than other types of nanoparticles. Their physicochemical properties, such as easy manipulation of particle size, surface charge, and the availability of many functional groups that can be exploited for conjugating different biomolecules and targeting ligands [26], make polymeric nanoparticles attractive carriers. Polymeric nanoparticles have been used to address the major limitations of the gene delivery process, such as poor cell uptake, lysosomal degradation, poor transfection efficiency, and off-target effects [27–29]. In this review, we discuss different polymeric nanoparticles for gene delivery in lung cancer. The most commonly explored polymer-based nanoplatforms for gene delivery are synthetic (polylacticacid-co-glycolic acid) or natural (chitosan) polymer nanoparticles, polymeric micelles, and dendrimers (Fig. 1).

### PLGA

Polylactic-co-glycolic acid (PLGA) is a co-polymer of lactic acid and glycolic acid bonded by ester linkages and is biocompatible and biodegradable [30]. PLGA was approved as a drug delivery carrier for parenteral administration by the FDA and European Medicine [31]. PLGA nanoparticles can be synthesized through different methods. One of most popular methods is emulsion-solvent evaporation to obtain sphere-shaped nanoparticles [32]. The therapeutic agents, drugs, or genes are either encapsulated inside the core or adsorbed onto the nanoparticle surface [33]. PLGA-based nanoparticles protect siRNA from degradation and overcome the cellular barriers to facilitate efficient transfection [34, 35]. In addition, hydrophilic and/or hydrophobic drugs can be encapsulated inside PLGA nanocapsules for co-delivery of chemotherapeutics and nucleic acid therapeutics [36, 37]. The PLGA nanoparticles can be internalized into cells through fluid phase pinocytosis and clathrin-mediated endocytosis [38]. Since PLGA nanoparticles exhibit a negative surface charge, they can be modified with hydrophilic and cationic polymers, such as PEI and chitosan, for electrostatic adsorption of gene molecules.

To adsorb siRNA, negatively charged PLGA nanoparticles are often modified with a strong cationic polymer, PEI. In a typical study, PEI-modified nanoparticles were used for co-delivery of paclitaxel and a siRNA therapeutic for cancer therapy [39]. Paclitaxel, the hydrophobic anti-cancer drug, was encapsulated in PLGA nanoparticles, and stat3 siRNA was adsorbed onto the PLGA-PEI nanoparticles through electrostatic interaction. The researchers used fluorescence measurements to confirm that stat3 siRNA and PTX were delivered simultaneously to A549 lung cancer cells via PLGA-PEI nanoparticles. Stat3 was activated in lung cancer cells (A549 and A549/T12), and siRNA-based silencing of the stat3 gene using PLGA-PEI-TAX-S3SI nanoparticles rendered cancer cells more sensitive to PTX and produced more cellular apoptosis than did PLGA-PEI-TAX [39].

Cationization of PLGA nanoparticles is also possible with biocompatible chitosan, a carbohydrate polymer. Chitosan-modified PLGA nanoparticles were successfully used to deliver antisense oligonucleotides (DNA/RNA) to lung cancer cells in a recent study [40]. The chitosan-modified PLGA nanoparticles were 130 nm in size with an adjustable positive surface charge. Antisense oligonucleotides and 2′-*O*-methyl-RNA (OMR) for the human telomerase gene were electrostatically bound to chitosan-PLGA nanoparticles, and efficient cellular uptake was observed [40]. The chitosan content, binding efficiency, stability, and cell uptake efficiency of the chitosan-PLGA nanoplexes were evaluated for OMR delivery in a follow-up study by the same group [41]. The researchers observed that the cellular uptake and transfection efficiency of OMR was dependent on the chitosan content in the nanoparticle. The nanoparticles were non-toxic and efficiently inhibited telomerase activity.

PLGA nanoparticles generally exhibit good stability in aqueous systems. However, prolonged stability is a prerequisite for a good drug delivery system. Stabilizers, such as Pluronic F68 or PVA, are common choices for preparation of PLGA nanoparticles, but are known to be toxic [42, 43]. In a recent study, carbopol, a rheology-modifying polymer, was used to stabilize a PLGA nanoparticle system carrying DNA [44]. Carbopol was less toxic than Pluronic F68 when used as a stabilizer for PLGA nanoparticles. These PLGA nanoparticles showed more than 80% DNA-binding efficiency with optimal carbopol concentration and protected DNA from enzymatic degradation. Moreover, these carbopol-stabilized PLGA nanoparticles showed higher transfection efficiency in A549 cells compared with Pluronics F68-stabilized nanoparticles or naked DNA, with DNA transfection efficiency similar to that of Lipofectamine 2000. Thus, the choice of stabilizers for PLGA nanoparticles is an important consideration when designing stable and less toxic nucleic acid drug delivery systems.

### PEI

**Fig. 2:**
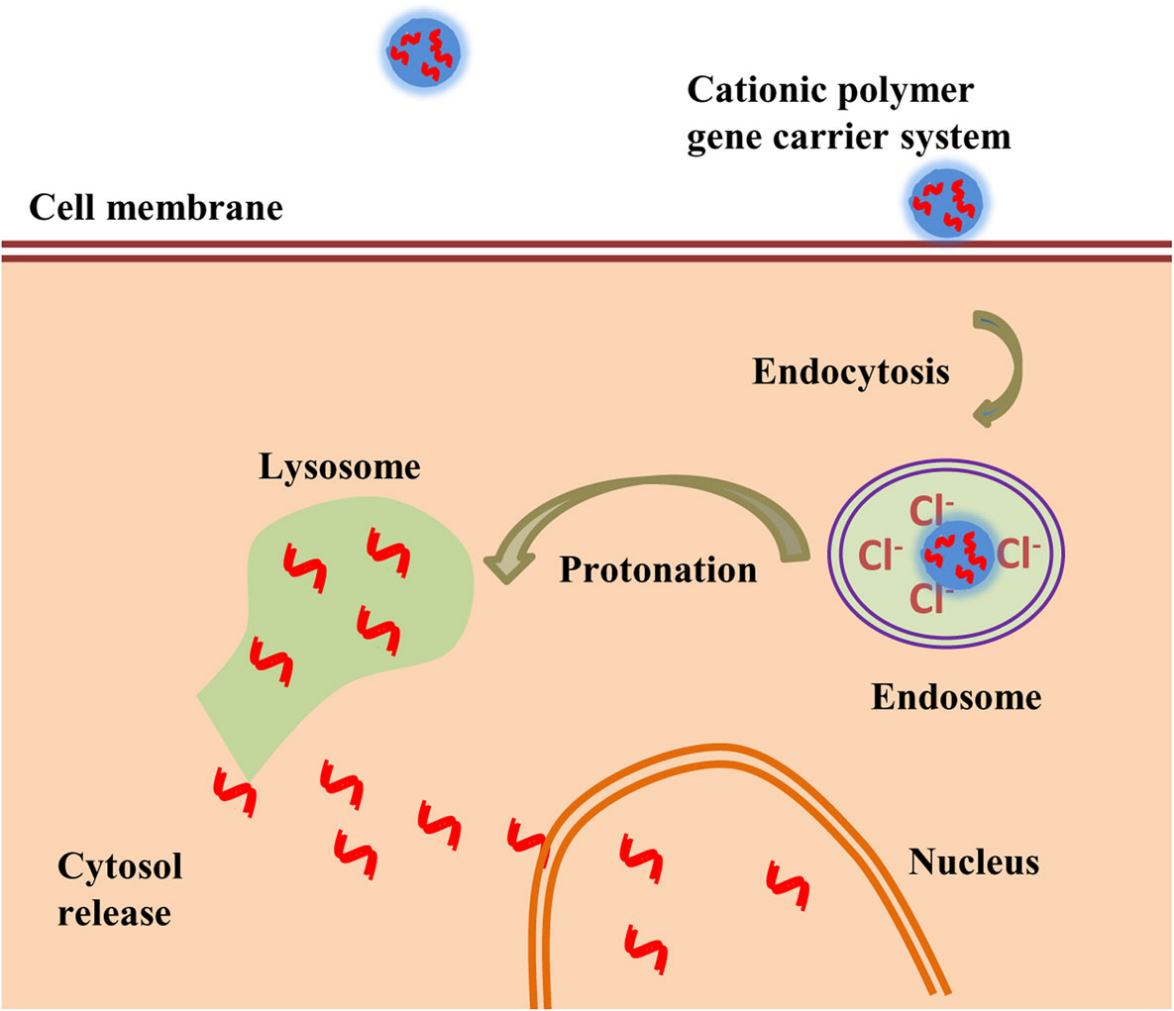
Schematic representation of cationic nanoparticles exploiting the proton sponge effect for gene delivery in cancer cells

Polyethylene imine (PEI) is a cationic polymer of ethylenediamine monomers that have linear and branched conjugations with water-soluble and protonatable amino groups. PEI is used prominently as a gene delivery vehicle because it exhibits high gene transfection efficiency [45]. Since PEI has a high positive charge density, it can form stable complexes with negatively charged nucleic acids through electrostatic interactions. The cationic amine groups of PEI can bind to anionic cell surface residues and internalize into cells through endocytosis. They have the ability to rescue the nucleic acid therapeutics from degradation in the endo-lysosomal compartment by inducing a proton sponge effect [46–48]. In the proton sponge effect, the protonatable amine groups in the cationic polymers of the gene carrier resist the acidification of endosomes during their maturation. This phenomenon leads to continuous proton pumping into the endosomes and reduces the pH inside the endosomal compartment resulting in passive entry of chloride ions and consequently excess influx of water. Finally, the endosomes swell and rupture to release their contents into the cytoplasm (Fig. 2).

The PEI polymer can be easily modified with other polymers to improve the stability and therapeutic efficacy, to allow conjugation of targeting molecules for specific delivery, and to encapsulate chemotherapeutics for co-delivery [49]. Mattheolabakis et al. [50] synthesized a PEI-based polymer complex that included HA and polyethylene glycol (PEG; HA-PEI/AH-PEG). The cationic PEI, anionic HA, and siRNA formed a polyplex by simple mixing. This polyplex showed good siRNA encapsulation and transfection efficiency. Survivin-silencing siRNA pre-treatment in A549/DDP cells reportedly improved the cytotoxic activity of the CDDP-C6 and CDDP-C8 compounds. Further, the co-treatment of the survivin-silencing siRNA and CDDP-C8 induced more tumor growth inhibition than did CDDP alone in a model of CDDP-resistant lung cancer [50].

A different strategy utilized PEI grafted with another cationic polymer poly-l-lysine (PLL; PLL-alkyl-PEI) to reduce the cytotoxicity of individual polymers and improve gene delivery efficiency. The shRNA delivery efficiency of PLL-alkyl-PEI was evaluated by aptamer modified or un-modified nanoplexes, which exhibited acceptable cytotoxicity and resulted in a 1.8–5-fold increase in transfection efficiency in lung cancer cell lines [51]. Similarly, numerous studies have shown the ability of PEI and PEI-grafted copolymers to successfully deliver genes to cancer cells [52–54]. Based on these studies, low-molecular-weight PEI would be a better option for gene delivery applications because of its low toxicity.

The abundant presence of free amino groups makes PEI an attractive polymer for conjugation of various ligands to improve the transfection efficiency or targeted gene delivery. In a typical example, PEI was covalently conjugated with cell-penetrating oligopeptide TAT (related to the protein transduction domain of HIV-1) through a heterobifunctional PEG spacer, resulting in a polyplex named TAT-PEG-PEI [54]. This TAT-PEG-PEI polyplex improved DNA reporter gene complexation and protection as well as stability against polyanions, AlveofactR, bronchial alveolar lining fluid, and DNase. Compared with PEI, the TAT-PEG-PEI polyplex showed better DNA transfection efficiency and lower toxicity in an A549 lung cancer model in vitro and in vivo [55].

A recent report showed that when sorbitol diacrylate (SDA) was crosslinked with low-molecular-weight PEI to form a poly-sorbitol-mediated transporter (PSOT), the siRNA transfection was significantly improved in vitro and in vivo. Here, low-molecular-weight PEI, which reduced the carrier’s cytotoxicity, was used in PSOT synthesis. When PSOT was complexed with osteopontin (OPN) siRNA, it efficiently silenced OPN protein, which was overexpressed in A549 and H460 NSCLC cell lines, and suppressed tumor growth in two mouse xenograft tumor models [56].

### Chitosan

Chitosan is a natural, polysaccharide-based polymer derived from chitin [57]. It is widely used in many biomedical applications because of its biocompatibility and biodegradability and can be easily modified with targeting ligands [58]. Chitosan is one of the best candidates for nucleic acid delivery because of its amine groups with a positive surface charge. Protonation of the chitosan amine groups occurs at a pH below its p*K*a value of 6.6. This strong pronation of amines enhances the electrostatic interaction between chitosan and nucleic acids to form complexes of nanoscale dimensions [59, 60]. The optimal nitrogen-to-phosphate charge ratio (N/P) of chitosan to DNA/siRNA molecules also supports strong condensation, which protects the nucleic acid therapeutics from nuclease digestion while in the circulation. The percentage of deacetylation as well as molecular weight of chitosan also affects the DNA/siRNA transfection efficiency [61, 62].

Taetz et al. [63] demonstrated that different amounts of cationic chitosan were complexed with PLGA through emulsion-solvent evaporation to form chitosan/PLGA nanoparticles (CPNPs). These CPNPs were used to deliver an antisense 2′-*O*-methyl-RNA (2OMR) directed against an RNA template of human telomerase. They found that the binding efficiency and complex stability of CPNP with 2OMR were high in water and correlated well with the chitosan content of particles, but were weak in physiologically relevant medium (PBS and RPMI cell culture medium). Their flow cytometry analysis revealed that the uptake of 2OMR into A549 lung cancer cells was considerably higher when transfecting with CPNP, and the efficiency in inhibiting telomerase activity was dependent on the amount of chitosan used in the complex.

Variations in chitosan molecular weight (*M*_w_) and the degree of deacetylation (DD) also affect the gene transfection efficiency, as reported in a recent study [64]. SiRNA (for EGFP) formulations prepared with different *M*_ws_ (∼10, 64.8–170, and 114–170 kDa) of chitosan (80% DD) showed silencing efficiencies of 0, 45–65, and 80%, respectively, in EGFP-enhanced H1299 human lung carcinoma cells. Notably, high *M*_w_ and high DD chitosan formed stable 200-nm nanoparticles and displayed the highest gene silencing efficiency (80%) at an N/P ratio of 1:150.

To improve the delivery of siRNA and overall transfection efficiency, chitosan was grafted to PEI (CHI-g-PEI). The low Mw PEI combined with chitosan grafting resulted in a strong cationic copolymer that stably complexed with siRNA. Compared with PEI (25 kDa) as a siRNA delivery system, the chitosan-g-PEI system silenced EGFP siRNA ~2.5 times more efficiently. This copolymer system also delivered onco-protein-targeted Akt1 siRNA with high efficiency. Silencing of Akt1 protein with the siRNA/CHI-g-PEI complex resulted in significant reductions in lung cancer cell survival, proliferation, and metastasis [65].

The presence of many functional groups in chitosan polymers is a great advantage for ligand modification for targeted gene delivery. A recent study reported the synthesis of EGFR-targeted chitosan nanoparticles to deliver siRNA-targeting Mad2 (siMad2), alone or in combination with cisplatin, toward drug-resistant A549 cells. Efficient silencing of the Mad2 gene improved the toxicity of cisplatin by overcoming the cisplatin resistance in a human lung adenocarcinoma xenograft model [66]. Another study reported the modification of chitosan with folate to target folate-receptor-overexpressing HeLa and OV-3 cell lines. The findings suggest that folate modification not only improved the transfection efficiency, but also reduced the toxicity of chitosan nanoparticles [67]. Several examples of ligand-conjugated, chitosan-based gene delivery systems are available in the literature, and many of these chitosan-based systems are promising candidates for cancer gene therapy [68].

### Dendrimers

Dendrimers are polymer-based, synthetic, highly branched, monodisperse, and spherical nanomaterials of less than 10 nm in size [69]. These dendrimers have peripheral functional groups that can be functionalized with chemotherapeutics, targeting molecules, and other bioactive molecules; the interior cavities can also be encapsulated with hydrophilic and hydrophobic molecules for combination therapy and imaging [70]. Poly- (amidoamine; PAMAM) and poly (propeleneimine; PPI)-based nanoparticles are extensively used as gene delivery vehicles because of their biocompatibility and biodegradability [71, 72]. The amine groups in PAMAM and PPI confer a cationic charge onto their surfaces, which can participate in encapsulation of DNA/siRNA gene molecules through electrostatic interactions. Dendrimer-based nanoparticles enhance protection from enzyme degradation and improve the cellular uptake of DNA/siRNA [73, 74].

Taratula et al. [75] reported a PPI-dendrimer system complexed with siRNA. The siRNA-PPI system was then crosslinked with dithiol cross-linker followed by a PEG polymer coating to improve the steric stability and circulation time. To target these nanoparticles to specific cancer cells, luteinizing hormone-releasing hormone (LHRH) peptide was conjugated through the distal end of the PEG polymer. These layer-by-layer PPI targeted nanoparticles allowed specific tumor uptake and target gene silencing in LHRH receptor-positive A549 human lung cancer cell lines. In vivo biodistribution also showed specific delivery of siRNA to tumors, which may reduce the side effects in normal tissues [75].

The potential of the PAMAM dendrimer for targeted gene delivery has been demonstrated by many researchers. In a typical study, PAMAM G5.0 dendrimer with aptamer modification through a PEG complex (PAM-Ap) was used to deliver pMiR-34a against A549 human lung cancer cells [76]. In NSCLC cells, this PAM-Ap/pMiR-34a nanoparticle showed improved cellular uptake and efficient gene transfection compared with non-targeted nanoparticles, resulting in regulation of target genes (BCL2 and p53) and anti-tumor effects. Further, Biswas et al. [77] developed a lipid-modified, dendrimer nanoparticle, triblock co-polymeric system, poly(amidoamine) dendrimer (generation 4)-poly(ethylene glycol)-1,2-dioleoyl-*sn*-glycero-3-phosphoethanolamine (G(4)-D-PEG-_2K_-DOPE). In this study, a PAMAM-G4 dendrimer was used as a cationic source for siRNA condensation. Then, G(4)-D-PEG-2K-DOPE was incorporated into the PEG-_5K_-PE micelles. This lipid-modified micellar nanocarrier stably carried siRNA and showed serum protection, efficient cellular uptake, and transfection of siRNA in A549 lung cancer cells [77]. Thus, dendrimers are excellent alternative carriers for gene therapeutics in lung cancer treatment.

### Polymer Micelles

Micelles are spontaneous self-assemblies of amphiphilic copolymers in a spherical shape of ~100 nm in water. The hydrophobic groups, which can hold hydrophobic drugs, are on the inner side of micelles, whereas the outer shell containing hydrophilic polymers can interact with different types of bioactive molecules, such as targeting moieties and DNA/siRNA [78, 79]. Micelles are promising gene delivery systems because of their excellent controlled release and tissue-penetrating ability. The electrostatic interaction between micelles and DNA/siRNA molecules results in the formation of a micelle-gene complex [80]. The advantage of micelles is that the copolymers with tumor-targeted, stimuli-sensitive release properties, like acid- or glutathione-sensitive cleavage bonds, can be chosen for site-specific controlled delivery [81, 82]. Sun et al. [83] engineered an acid-sensitive Dlinkm group copolymer micelleplex based on the self-assembly of PEG-Dlinkm-R9-PCL polymers that interacted with siRNA to form Dm-NP/siRNA through electrostatic bonds. This Dm-NP/siRNA protected siRNA in serum, increased its circulation time, and enhanced the uptake of siCKD4 in A549 lung tumor cells. Further, Dm-NP/siRNA improved the gene silencing efficiency and anti-tumor activity in vivo through pH-controlled delivery of therapeutic siRNA [83]. Another group developed a polyanion micellar system (PIC micelle) with tumor targeting and endosomal disruption abilities for efficient delivery of its RNAi payload [84]. The polypeptide PAsp(DET-CDM/DBCO) created from acid-labile carboxydimethyl maleate (CDM) and dibenzylcyclooctyne (DBCO) can tune the net change in the extracellular environment against lung cancer cells and was used to modify PIC micelles for pH-responsive cellular delivery of siRNA. Targeted, efficient, and less cytotoxic siRNA therapy was achieved using this modified PIC micellar system.

### Polyspermine

Polyspermine is a dynamic and biologically responsive polymeric system that can form nanoparticles by complexation with nucleic acid therapeutics. A recent report suggests that biocompatible polyspermine polymer nanoparticles are an excellent alternative plasmid DNA delivery system for lung cancer treatment [85]. Spermine has the ability to deliver shRNA in a mouse model of lung cancer. The researchers synthesized glycerol triacrylate and spermine (GT-SPE) through a Michael addition reaction. GT-SPE/DNA nanoparticles showed less toxicity and more transfection efficiency, and they were safe for use in vitro and in vivo, compared with a PEI transfection system. The aerosol-delivered GT-SPE/small hairpin Akt1 (shAkt1) complex suppressed lung tumorigenesis in a Kras-LA1 mouse model of lung cancer by inducing cell cycle arrest and apoptosis through the Akt signaling pathway [85].

Later, the same group synthesized a spermine (SPE) and PEG diacrylate (SPE-alt-PEG) copolymer with a Michael-type addition reaction [86]. They made a complex of SPE-alt-PEG polyspermine with plasmid DNA, which showed good DNA protection. The SPE-alt-PEG copolymer reportedly has low cytotoxicity and showed higher DNA transfection efficiency than 25-kDa PEI (PEI 25K). These SPE-alt-PEG/GFP complexes were administered as aerosols and accumulated in the lungs with no apparent toxicity. SPE-alt-PEG/GFP has been shown to deliver the Pdcd4 gene to the lungs, causing significant reductions in tumor size and tumor number in a Kras-LA1 mouse model of lung cancer, when compared with PEI 25K alone.

Table 1 summarizes some of the recently explored polymer nanoparticle systems that have been used for gene delivery in lung cancer. The development of newer polymeric systems with excellent safety properties and target specificity for gene delivery is an important step toward the exploitation of their potential to the fullest in lung cancer therapy. However, rapid translation of many of these polymeric systems to the clinic should surpass the time-consuming clinical trials and FDA regulations for safety and potency. Improvement of existing polymeric systems with a good safety profile for gene delivery is therefore the focus of many researchers worldwide for cancer therapy.

**Table 1:** Examples of polymers used for nanoparticle formulation for gene delivery in lung cancer therapy

Polymer(s)	Gene therapy tool(s)	Lung cancer type/model	References
PLGA	siRNA	A549	[39]
PLGA	Plasmid DNA	A549	[44]
PLGA/Chitosan	Oligonucleotides/siRNA	A549	[41]
PEI/AH-PEG	siRNA	A549	[50]
PEG-PEI	Plasmid DNA	A549	[55]
PEI-PSOT	siRNA	A549	[56]
PLL-alkyl-PEI	shRNA	–	[51]
Chitosan	2′-*O*-methyl-RNA	A549	[63]
Chitosan	siRNA	A549	[66]
Chitosan	siRNA	H1299	[64]
Chitosan-graft-PEI	siRNA	A549	[65]
Poly (propyleneimine) (PPI) dendrimers	siRNA	A549	[75]
Poly (amidoamine) (PAMAM)	Plasmid DNA	A549	[76]
Poly (amidoamine) (PAMAM)	siRNA	A549	[77]
Polymer micelles	siRNA	A549	[83, 84]

## Polymeric Nanoparticles for Receptor-Mediated Gene Delivery to Lung Cancer

Targeting of nanoparticle-based gene delivery systems has been explored for improved uptake of nucleic acid therapeutics at the tumor site and to prevent undesirable off-target effects in healthy tissues. While passive targeting explores the enhanced permeation and retention (EPR) effect in the tumor microenvironment, active targeting involves the ligand-receptor interaction mechanism between the nanoparticle carrier and cancer cells. Since the active targeting mechanism is known to be more efficient in site-specific delivery of the nucleic-acid payload, the following section presents different strategies for active gene delivery using polymer nanoparticles in lung cancer therapy. Tumor-specific ligands can be conjugated or decorated onto the polymer nanoparticle surface for targeted delivery of nucleic acid payloads [87, 88].

**Fig. 3:**
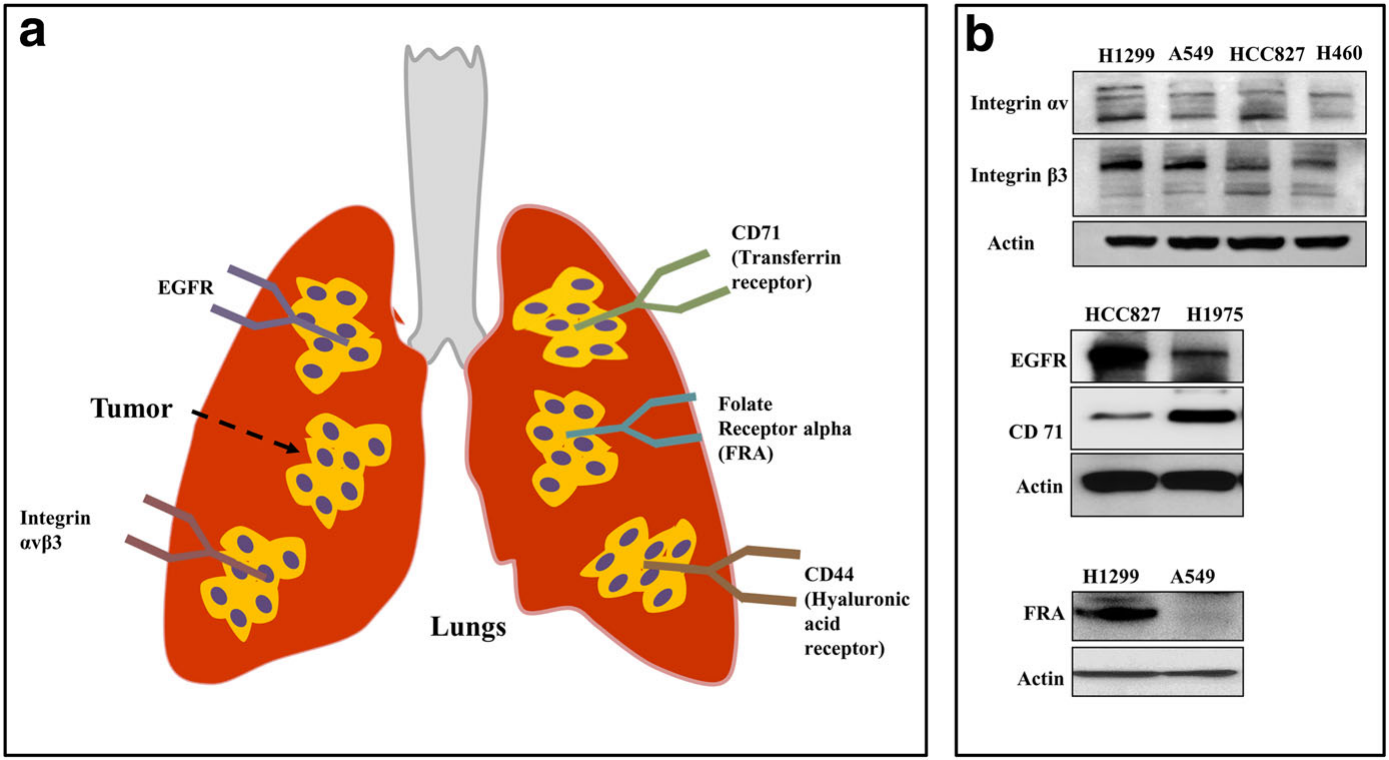
**a** Schematic representation of different cell surface receptors that are overexpressed in lung cancer that has been harnessed for targeted gene delivery using nanoparticles. **b** Western blots show the differential expression cell surface receptors among different lung cancer cell types

The targeted delivery efficiency depends on the ligand concentration and density, ligand-receptor binding affinity, surface charge, and stability of the polymeric nanoparticles [89, 90]. The targeting ligand specifically interacts with cell surface receptors that are overexpressed in lung cancer cells. Transferrin receptor, FRA, EGFR, integrins, and CD44 are common receptors that have been explored for targeted nanoparticle-based gene delivery in lung cancer (Fig. 3). Small molecules, proteins, antibodies, and antibody fragments have been used to target the receptors by functionalizing the polymeric nanoparticles on their surfaces [91].

### Folate Receptor Alpha

Folate receptor alpha and cell surface glycoprotein are members of the folate receptor family, which facilitates folate transport into cells [92]. FRA specifically interacts with folic acid and methotrexate and regulates the cellular uptake of folates and cell growth. Folate-conjugated nanoparticles enter cells predominantly via FRA-mediated endocytosis. Certain cancer cells, including lung cancer tumors, express high levels of FRA because of their epithelial origin [93, 94]. A significant number of lung cancer types, including adenocarcinoma, bronchioloalveolar carcinoma, and large-cell carcinoma, express detectable levels of FRA [95]. However, receptor expression levels may differ between the cancer types. For instance, 74% of lung adenocarcinomas expressed FRA, whereas only 13% of squamous cell carcinomas expressed FRA [96]. In one of our studies, we reported that lung cancer cells overexpressed FRA receptors compared with normal lung cells, indicating a clear differential expression of FRA between normal and lung cancer cells, which could be utilized for active targeting by nanoparticle gene delivery systems [97].

Researchers reported that polyspermine nanoparticles, when conjugated with folic acid ligand, resulted in efficient targeted gene delivery in FRA-overexpressing lung cancer in vitro and in vivo [98, 99]. Folic acid (FA) molecules were incorporated into polyspermine nanoparticles through either an acylation reaction [98] or amidation reaction [99]. Recently, the use of FA-conjugated chitosan-graft-PEI (FC-g-PEI) for small hairpin RNA (shRNA) delivery to lung cancer cells via an imine reaction was reported. The FC-g-PEI showed stable shRNA condensation and protection from DNase. This targeted gene carrier improved the lung tumor cellular uptake in vitro and in vivo, with reduced toxicity and significantly higher transfection efficiency than its non-targeted counterpart [100]. Thus, FRA targeting has emerged as an attractive active delivery strategy for polymer nanoparticle-assisted gene therapy for cancer.

### RGD

Integrins are heterodimeric transmembrane receptors that contain 18 α and 8 β subunits. In lung cancer, the composition of α and β subunits determines the tumor subtype, and integrin expression is heterogeneous [101, 102]. In lung cancer, integrin expression plays a behavioral and developmental role that influences patient survival [103]. Guo et al. [104] reported overexpression of integrins in a panel of different lung cancer cells, including A549, Calu-1, H1650 and DMS-53 cells, and specimens obtained from patients [104]. Expression of integrin subtypes is cell type dependent. However, α5 and β1 integrin expression was found in all types of lung cancer cells and patient specimens verified. Reports also suggest that αvβ3 integrin overexpression is characteristic to many lung cancer cell types and in tumor endothelium and plays a major role in angiogenesis [104, 105].

Among all integrins, the αvβ3 receptor was the best choice for targeted delivery of therapeutics into lung cancer. This receptor can be specifically targeted using RGD (Arg-Gly-Asp) peptide. The RGD peptide (linear or cyclic) should be conjugated on the surface of nanoparticles for preferential binding with αvβ3 receptors for site-specific delivery of the gene payload [105, 106].

Integrin αvβ3 receptor targeting has been extensively studied for targeted delivery of RGD-conjugated nanoparticle imaging agents for tumor diagnostics. A recent study successfully explored an RGD-peptide-conjugated PEGylated ^64^Cu-DOTA-PEG-E[c(RGDyK)]_2_ polymer-based system for positron emission tomography (PET) imaging for lung cancer. The researchers obtained clear lung tumor images by RGD-based targeting of the imaging agent, while the αvβ3 targeting permitted reduced nonspecific accumulation in normal lung and heart tissue [107].

Ragelle et al. developed a PEG-grafted chitosan-PEI nanoparticle surface decorated with RGD peptide ligands or RGD peptidomimetic (RGDp) for αvβ3-receptor-targeted gene delivery in lung cancer cells [108]. The RGD-modified nanoparticles with GFP siRNA showed two-fold higher cellular uptake than RGDp and demonstrated enhanced endo-lysosomal escape that resulted in 90% EGFP gene silencing in human cell lung carcinoma H1299 cells. It was noted that the cellular uptake and GFP silencing efficiency of chitosan-PEI/siGFP was dependent on the RGD surface concentration.

To evaluate a novel docetaxel-deslorelin derivative, Sundaram et al. [109] synthesized an RGD-conjugated nanoparticle for targeted delivery in lung cancer in vitro and in vivo. The RGD-conjugated PLGA nanoparticle containing docetaxel-deslorelin (D–D), a luteinizing hormone-releasing hormone (LHRH) agonist, was co-administered with anti-VEGF intraceptor plasmid (RGD-Flt23k-NP), which improved the therapeutic efficiency by VEGF inhibition in vivo in a H1299 xenograft mouse model significantly more than did individual targeted therapies or docetaxel alone [109]. RGD-assisted delivery thus enhanced the neovasculature-targeted combination therapy of Flt23k-NP and D-D.

### EGFR

Epidermal growth factor receptor is a receptor tyrosine kinase protein on the cell surface and is involved in cell growth, division, and proliferation [110, 111]. Overexpression of EGFR has been reported in different human malignancies, including lung tumors [112]. Some reports have shown that EGFR expression in lung cancer is related to poor chemosensitivity and a reduced survival rate [113, 114]. EGFR expression levels vary in different types of NSCLC cells, normal lung fibroblasts, and normal human bronchial epithelial cells. Most lung cancer cells expressed higher levels of EGFR than did normal cells [115]. Thus, EGFR expression in lung cancer cells has been explored for targeted gene delivery using nanoparticles. EGFR antibody or affibody can be used for EGFR-targeted delivery of therapeutics to lung cancer cells [116]. The targeting EGFR molecules can be conjugated on the surface of the nanoparticles through covalent or non-covalent interactions.

In a recent report, an EGFR receptor-targeted chitosan nanoparticle carrying siRNA was developed for Mad2 (mitotic checkpoint that induces cell death) gene silencing in A549 lung cancer cells. The targeted chitosan nanoparticles showed complete silencing of the Mad2 gene, whereas non-targeted nanoparticles showed significantly less silencing [117]. A bio-distribution study using EGFR-targeted chitosan nanoparticles showed six-fold higher tumor targeting efficiency than non-targeted nanoparticles in lung cancer tumor models [118]. In a different study, EGFR-targeted chitosan nanoparticles successfully delivered a combination of cisplatin and Mad2 siRNA in cisplatin-sensitive and -resistant in vivo lung cancer models. Cisplatin-resistant tumors showed improved tumor inhibition with EGFR-targeted combination therapy, which showed reduced toxicity to normal tissues [119].

### CD44

CD44 is a cell-surface integral membrane glycoprotein that functions as a receptor for hyaluronate and plays an important role in regulating tumor growth, metastasis, and drug resistance [120]. Reports have shown that the overexpression of CD44 indicates poor prognosis [121]. This glycoprotein is involved in different functions, such as cell adhesion and migration, and interacts with cell-matrix glycon [122]. CD44 expression is characteristic of different cancers, including lung cancer. Among lung cancers, NSCLC tumors express higher levels of CD44 than do SCLC. Hyaluronic acid specifically targets the CD44 receptor expressed in cancer cells; this specific interaction can be exploited for targeted delivery of therapeutics through conjugation of HA on the surface of nanocarriers [123, 124].

Ganesh et al. [125] introduced an HA-conjugated HA-PEI/PEG nanoparticle system for CD44-targeted delivery of siRNA to lung cancer cells. They screened many HA-functionalized lipids and polyamines for optimal siRNA encapsulation for therapeutic delivery. The CD44-receptor-mediated endocytosis of the HA-PEI/PEG-siRNA complex was confirmed by blocking the CD44 receptors with free soluble HA incubation before targeted nanoparticle incubation, resulting in reduced receptor-mediated endocytosis. The targeted HA-PEI/PEG nanoparticles showed enhanced cellular uptake and specific gene knockdown in vivo in sensitive and resistant A549 lung cancer tumors and metastatic tumors [125]. In a separate study, they evaluated the CD44-targeting, HA-based self-assembling nanosystems for delivery of a combination of cisplatin and siRNA(s) to overcome multidrug resistance in NSCLC. The targeted combination therapy significantly increased tumor growth inhibition through the synergistic therapeutic activities of cisplatin and siRNA, which increased the tumor growth inhibition from 30 to 60% by chemosensitizing cisplatin-resistant tumors [126].

In a different approach, dual-targeted HA-modified nanoparticles were used in the genetic transformation of tumor cells to manipulate the exosomal content secreted by tumor cells [127]. In this proof-of-concept study, CD44 and EGFR receptors overexpressed in SK-LU-1 lung cancer cells were explored for dual-targeted delivery of wild-type (wt-) p53 and microRNA-125b (miR-125b) expressing plasmid DNA via HA-PEI/HA-PEG. Strikingly, they observed the transgene expression of both p53 and miRNA-125b in the secreted exosomes, which mediated the macrophage repolarization toward an antitumor phenotype. Exosomes secreted by HA-nanoparticle/pDNA-transfected SK-LU-1 cells displayed reprogrammed micro-RNA profiles and activated p53-mediated apoptosis signaling pathways. Although the in vitro results are promising, in vivo studies are required to understand the effect of macrophage repolarization induced by these SK-LU-1-generated exosomes in tumor growth suppression and oncogenesis [127]. Taken together, these findings indicate that HA is an important targeted delivery ligand for polymer nanoparticle-mediated gene delivery toward CD44-expressing lung cancers.

### Transferrin

Transferrin receptor (TfR; CD71) is a type II transmembrane glycoprotein that is involved in cellular transport of iron and cell growth regulation [128, 129]. The TfR monomer contains a large extracellular C-terminal domain, a single-pass transmembrane domain, and a short intracellular N-terminal domain. A study revealed that 76% of lung adenocarcinomas and 93% of lung squamous cell carcinomas were positive for transferrin receptor, whereas normal lung tissues showed negligible expression [130]. Another study reported higher cell-associated TfR expression in bronchioalveolar lavage (BAL) of patients with NSCLC than in BAL of patients with chronic obstructive pulmonary disease (COPD) [131].

We reported that lung cancer cells express higher levels of transferrin receptors than do normal lung cells [132]. The higher expression levels of TfR in lung cancer can be explored for targeting gene delivery through receptor-mediated endocytosis [133]. However, there is limited literature pertaining to the development of TfR-targeted drug/gene delivery systems for lung cancer, probably because of high endogenous transferrin concentrations in plasma and high TfR expression in the blood-brain barrier. Nevertheless, studies show that the TfR-targeted delivery of theranostic agents and nanoparticle drug carriers toward brain malignancies is feasible [134]. To target transferrin receptors, the polymer nanoparticle surface should be functionalized with TfR-specific protein or antibody.

A typical example showed that transferrin-decorated, lipid-based nanostructured material (NLC) loaded with PTX and plasmid DNA was used to treat NCI-H460 human lung cancer cells. Tf was decorated onto NLC by a PEGylated Tf conjugate (Tf-PEG-PE). It was internalized by lung cancer cells via TfR-mediated endocytosis, resulting in successful transfection of delivered pDNA combined with co-delivered PTX. In vivo studies in a mouse tumor model demonstrated that TfR targeting is a feasible strategy for nanoparticle-assisted co-delivery of anti-cancer drug and gene therapeutics for the treatment of lung cancer [135]. Table 2 summarizes the literature reporting polymeric nanoparticles conjugated with ligands for receptor-mediated targeting in lung cancer.

**Table 2:** Summarized list of recently used polymer nanoparticles and their modifying ligand for targeted gene delivery in lung cancer

Targeting ligand/receptor	Polymer(s)	Nucleic acid therapeutic(s)	Lung cancer cell line	Reference(s)
Folic acid/FRA	Hyperbranched polyspermine	Plasmid DNA	A549	[98]
Folic acid/FRA	Polyspermine nanoplex modified with poly(ethylene glycol) (PEG) diacrylate (SPE-alt-PEG**)**	Plasmid DNA	A549	[99]
Folic acid/FRA	Chitosan-graft-PEI	shRNA	A549	[100]
RGD/Integrin αvβ3	Chitosan-PEI	siRNA	H1299	[108]
RGD/integrin αvβ3	PLGA	Plasmid DNA	H1299	[109]
EGFR ligand	Chitosan	siRNA	A549	[117, 119]
EGFR ligand	LPEI	siRNA	SPC-A1	[118]
Hyaluronic acid/CD44	HA-PEI/PEG	siRNA	A549	[125, 126]
Hyaluronic acid and EGFR ligand/CD44 and EGFR	Hyaluronic acid (HA)-based nanoparticles	miRNA and plasmid DNA	SK-LU-1	[127]

## Tumor Microenvironment in Lung Cancer Therapy

The tumor microenvironment (TME) in lung cancer is highly heterogenous with neovasculature formation and variable blood flow through immature blood vessels [136]. Lung carcinomas are closely associated with extracellular matrix (ECM), fibroblasts, tumor-associated macrophages (TAM), and other immune cells. This TME is essential for tumor initiation and growth, whereas in some cases it has tumor-suppressive roles [137]. Although the TME is critical for the pathogenesis of lung cancer, it can be exploited for therapeutic gain. Regions of acidosis and hypoxic conditions are important, which is common with lung cancer [138]. For example, the overexpression of hypoxia-inducible factor 1-alpha (HIF1α) under hypoxic conditions in tumors is a possible target for gene therapy [139]. HIF1α overexpression is involved in angiogenesis, cell survival, glucose metabolism, and invasion. Therefore, inhibition of HIF1α would impact the tumor growth and progression [140]. Further, the acidic environment in tumor cells can be exploited for pH sensitive gene delivery purposes. Polymers that are sensitive to pH changes are used in the construction of nanoparticles for gene delivery [141]. The acidic environment cleaves the pH-sensitive linkage and releases the nucleic acid molecules within the tumor milieu.

**Fig. 4:**
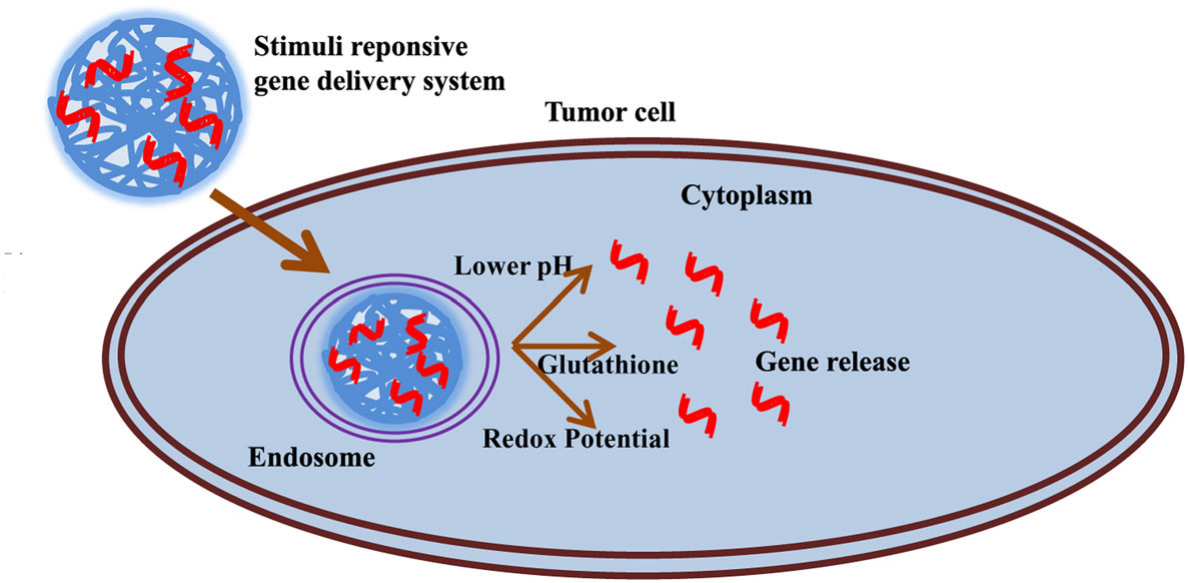
Tumor cell microenvironment assists in stimulus-responsive gene delivery

The concentrations of glutathione and other reducing substances are substantially increased in a tumor cells compared to surrounding normal cells [142]. This reducing environment in tumor cells is exploited for thiol-disulfide exchange reactions with glutathione molecules in cleaving disulfide bonds of nanoparticle-drug conjugates to facilitate the drug release [143]. Various nanoparticles have been designed with polymeric materials to explore the reducing environments in tumors for controlled release of gene therapeutics. A schematic representation of stimuli-responsive gene delivery using polymer nanoparticles sensitive to the tumor microenvironment is shown in Fig. 4. In a specific example, Tai et al. [144] constructed a stearyl-peptide cross-linked with disulfide bonds for the delivery of siRNA. This stearyl-peptide efficiently condensed siRNA to form a polyplex, and upon cellular entry it rapidly dissociated the siRNA into the cytoplasm to achieve good transfection efficiency. As mentioned elsewhere, the tumor microenvironment in lung cancer is characterized by overexpression of several different receptors in the cell surface. Targeted drug/gene delivery using nanoparticles toward lung cancer can be achieved by exploiting the ligand-receptor affinity.

Nanoparticle-based targeted gene delivery to lungs can be possible through different administration routes. Systemic administration is commonly practiced in chemotherapeutic intervention of lung cancer. Recent developments in pulmonary drug delivery strategies show promise in developing nanoparticle-based gene delivery systems for intrapulmonary lung cancer therapy [145]. The large surface area of the respiratory system, thin alveolar epithelium, rapid adsorption, high bioavailability, and enhanced drug uptake make pulmonary route administration optimal compared to systemic delivery. Moreover, this localized drug/gene delivery may minimize systemic side effects of the drug.

### Challenges in Using Polymeric Gene Delivery Systems In Vivo

The majority of gene delivery systems are cationic, and the density of this cationic charge is an important determinant of the toxicity of the nanocarrier materials. Positively charged polymers such as PEI, PAMAM, or PPI dendrimers have shown toxicity at the level of gene expression alterations. Such alternations are generally termed as carrier mediated “off-target effects”. Reports also suggest that even non-ionic polymers such as PEG and PEG-poly-glutamic acid block copolymers can also cause non-specific gene alterations [146]. Therefore, it is very crucial to study the effect of polymer materials to gene signatures prior to its in vivo evaluation. Another hurdle faced by polymeric gene carrier nanosystems is the interaction with serum proteins. Cationic polymers are highly prone to interact with negatively charged serum proteins, which lead to their rapid clearance by a reticuloendothelial system while in circulation. This reduces the accumulation of gene therapeutics in the desired site in the body. Engineering the nanoparticle surface with functional groups or biomimetic polymers can not only delay the RES uptake of polymer nanoparticles, but also enhance the uptake of therapeutic payload in the desired tissue. A biomimetic polymer is artificially made but imitates the biological system and fools the body’s defense mechanism for their capacity to recognize the polymer material as a foreign body. Thus, biomimetic polymers take advantages of their interaction with biological systems without causing any harm to the body. Many of the widely used polymers in drug or gene delivery such as polylactones, polypeptides, and dendrimers fall in this class of bioinspired polymeric materials. However, there are still challenges to overcome to meet clinical safety. A recent review by Dehaini et al. [147] has comprehensively reviewed the biomimetic strategies for nanoparticle-based drug delivery. In a nutshell, the polymeric nanosystems developed for drug/gene delivery should meet the clinical standard by undergoing stringent evaluation especially of the kind of genotoxicity, and the gene delivery systems should be controllable in their interaction with genes; a clear understanding of the transfection mechanism is important while testing novel gene delivery systems.

## Conclusion and Prospects

Over the last decade, advancements in nanoparticle-based delivery systems have brought safer, more effective new treatment approaches for lung cancer that have allowed for the rapid clinical translation of many drugs. As a relatively newer therapeutic modality for lung cancer, gene therapy has shown great potential. Among the nanoparticle carriers, polymer nanoparticles have substantial advantages, such as easy tunability of the physico-chemical properties to suit in vivo delivery of nucleic acids, a good safety profile, and the potential for targeted delivery, for gene delivery applications.

The problem of polymer material toxicity can be limited by adding safe co-polymers and by careful modification of polymers with non-immunogenic moieties. Various polymer nanoparticles have shown promise as future therapeutic carriers for lung cancer. The use of targeting moieties, such as small molecules or antibodies specific to receptors that are overexpressed in lung cancers, would add a new dimension to efficacious lung cancer treatment using these polymer-based nanoparticle gene delivery systems. The overexpression of cell surface receptors in lung cancer can be harnessed for targeted delivery of gene therapeutics using nanoparticles. Since most of these polymers can be easily modified by targeting ligands, the off-target effects of the drug or gene therapy cargo can be limited by site-specific delivery.

Polymeric nanocarriers are capable of carrying multiple therapeutics for cancer therapy; however, further advancements in this area are required. Using a multiple therapeutic combination enhances the complexity of the polymeric nanoparticle systems, which requires careful formulation, and evaluation of controlled release, physico-chemical, and structural characteristics.

Advancements in polymeric gene delivery systems are highly anticipated with the development of stimuli-responsive polymers in nanoparticle formulation. It is possible to deliver a drug in spatial-, temporal-, and dosage-controlled fashions using stimuli-responsive drug delivery systems. Carefully engineered stimuli-responsive polymers with biocompatibility are required to implement such gene/drug delivery systems that potentially deliver the bio-actives over pH changes, hydrolytic action, or supramolecular confirmation change. With major advances in understanding the tumor microenvironment and the knowledge of how drug delivery systems can affect the successful delivery of the payload to the desired site, rapid clinical translation of these polymer-based gene delivery systems could be possible in the future.
